# The Light Chains of Microtubule-Associated Proteins MAP1A and MAP1B Interact with α1-Syntrophin in the Central and Peripheral Nervous System

**DOI:** 10.1371/journal.pone.0049722

**Published:** 2012-11-13

**Authors:** Heike Fuhrmann-Stroissnigg, Rainer Noiges, Luise Descovich, Irmgard Fischer, Douglas E. Albrecht, Fatiha Nothias, Stanley C. Froehner, Friedrich Propst

**Affiliations:** 1 Max F. Perutz Laboratories, University of Vienna, Department of Biochemistry and Cell Biology, Vienna, Austria; 2 Department of Physiology and Biophysics, University of Washington, Seattle, Washington, United States of America; 3 INSERM U952, CNRS UMR 7224, Université Pierre et Marie Curie (UPMC) - Paris-06, Paris, France; Aix Marseille University, France

## Abstract

Microtubule-associated proteins of the MAP1 family (MAP1A, MAP1B, and MAP1S) share, among other features, a highly conserved COOH-terminal domain approximately 125 amino acids in length. We conducted a yeast 2-hybrid screen to search for proteins interacting with this domain and identified α1-syntrophin, a member of a multigene family of adapter proteins involved in signal transduction. We further demonstrate that the interaction between the conserved COOH-terminal 125-amino acid domain (which is located in the light chains of MAP1A, MAP1B, and MAP1S) and α1-syntrophin is direct and occurs through the pleckstrin homology domain 2 (PH2) and the postsynaptic density protein 95/disk large/zonula occludens-1 protein homology domain (PDZ) of α1-syntrophin. We confirmed the interaction of MAP1B and α1-syntrophin by co-localization of the two proteins in transfected cells and by co-immunoprecipitation experiments from mouse brain. In addition, we show that MAP1B and α1-syntrophin partially co-localize in Schwann cells of the murine sciatic nerve during postnatal development and in the adult. However, intracellular localization of α1-syntrophin and other Schwann cell proteins such as ezrin and dystrophin-related protein 2 (DRP2) and the localization of the axonal node of Ranvier-associated protein Caspr1/paranodin were not affected in MAP1B null mice. Our findings add to a growing body of evidence that classical MAPs are likely to be involved in signal transduction not only by directly modulating microtubule function, but also through their interaction with signal transduction proteins.

## Introduction

The vertebrate MAP1 family of microtubule-associated proteins consists of three members, MAP1A, MAP1B, and MAP1S. MAP1A and MAP1B are >300 kDA proteins and are expressed at high levels in the central and peripheral nervous system in the adult and during development, respectively [1]. MAP1S is smaller (120 kDa) and is ubiquitously expressed [2]. All three proteins share several defining features. They are synthesized as polyprotein precursors and are subsequently cleaved into a heavy and a light chain which bind to each other to form the respective MAP1 complex [1], [2]. Heavy and light chains of all MAP1 proteins contain structurally and functionally conserved domains that mediate heavy chain-light chain interaction, microtubule binding, and the potential to interact with F-actin [1]–[5].

The best characterized member of the MAP1 family is MAP1B, a 320-kDa protein which is expressed in the central nervous predominantly during development and in the peripheral nervous system throughout life [1], [6]. While originally thought to be expressed mainly in neurons, MAP1B was found to be expressed in Schwann cells [7] and oligodendrocytes [8]–[10] as well. Consistent with its expression in the nervous system, MAP1B deficient mice display defects in brain development [11]–[14]. In the peripheral nervous system, MAP1B deficiency results in a reduced number of large myelinated axons, the reduced thickness of myelin sheaths, and a decrease in nerve conduction velocity in the sciatic nerve [13].

In order to elucidate molecular mechanisms that might be involved in the function of MAP1B during development we performed a search for protein interaction partners using one of the domains conserved between MAP1A, MAP1B, and MAP1S as bait. Here we show that the COOH terminus of the light chain of MAP1B interacts with α1-syntrophin, a modular adapter protein associated with the dystrophin-glycoprotein complex (DGC) [15]–[18]. α1-syntrophin, a 58-kD protein highly expressed in the brain, belongs to a multigene family which consists of five isoforms α1, ß1 and ß2, γ1 and γ2. The syntrophins function by recruiting signaling molecules through their multiple protein interaction motifs. These consist of pleckstrin homology domains 1a, 1b, and 2 (PH1a, PH1b, PH2), a PDZ (postsynaptic density protein 95/ disk large/zonula occludens-1 protein homology) domain, and the syntrophin unique domain (SU). α1-syntrophin associates with the DGC in the plasma membrane of several cell types via direct binding of its PH2 and SU region to dystrophin, dystrobrevin or utrophin [19], [20]. The PDZ domain of α1-syntrophin binds to a variety of signaling molecules including sodium channels [21], [22], neuronal nitric oxide synthase [23]–[25], aquaporin-4 [26], [27] and serine/threonine kinases [28], [29]. Mice lacking α1-syntrophin display aberrations in neuromuscular synapses with undetectable levels of postsynaptic utrophin and reduced levels of acetylcholine receptor and acetylcholinesterase [30].

## Materials and Methods

### Ethics Statement

Tissues from mice were obtained in compliance with the Austrian law regulating the use of animals in biomedical research, Tierversuchsgesetz, BGBl. Nr. 501/1989 and BGBl. I Nr. 162/2005. The manuscript does not include experiments on live animals. The production and culling of mice in order to obtain tissues (as performed in this manuscript) does not require approval of the Austrian Ministry of Science and Research, the governmental body regulating the use of animals in biomedical research. Wild-type and MAP1B−/− mice were anesthetized and sacrificed by decapitation.

### Yeast 2-hybrid Screen and Recombinant Clones

**Figure 1:**
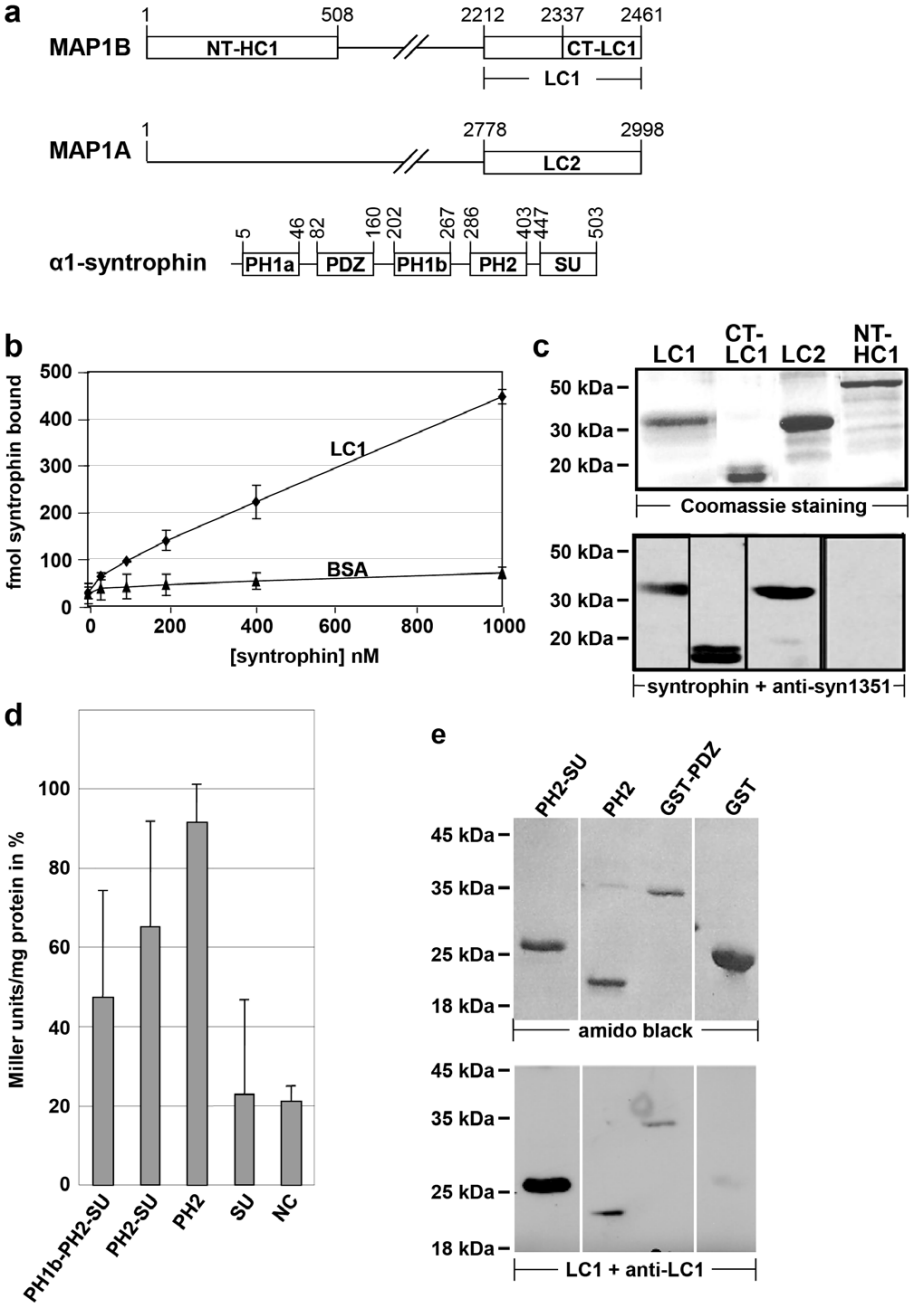
The light chains of MAP1B and MAP1A interact with α1-syntrophin. **a)** Schematic representation of MAP1B, MAP1A, and α1-syntrophin to indicate the relevant domains. The drawings are not to scale. Numbers indicate amino acid positions for rat MAP1A [71], [72], rat MAP1B (according to database entry XM_215469), and mouse α1-syntrophin [16], [32]. MAP1B and MAP1A are synthesized as polyprotein precursors which are proteolytically cleaved to yield heavy chains (HC1 and HC2, respectively) and light chains (*LC1* and *LC2*, starting at residue 2212 and 2778 of the polyprotein precursor, respectively). *NT-HC1*, NH_2_ terminus of HC1; *CT-LC1*, COOH terminus of LC1; *PH1a*, *PH1b*, and *PH2*, pleckstrin homology domains; *PDZ*, postsynaptic density protein 95/disk large/zonula occludens-1 protein homology domain; *SU*, syntrophin unique domain; **b)** Microtiter plates coated with 100 nM recombinant LC1 (*LC1*) or BSA (*BSA*) were overlaid with increasing concentrations of Eu^3+^-labeled recombinant α1-syntrophin. α1-syntrophin was found to bind specifically to LC1, whereas binding to BSA was weak and considered to be unspecific background of the assay. The values represent the mean ± standard deviation of three independent experiments. **c)** The indicated recombinant proteins were subjected to SDS-PAGE, stained with Coomassie brilliant blue or blotted onto nitrocellulose and probed with recombinant α1-syntrophin protein. For immunological detection of syntrophin bound to blotted proteins the pan-syntrophin antibody (anti-syn1351) was used. Syntrophin was found to bind to LC1, LC2 and the COOH-terminal half of LC1, but not to the NH_2_-terminal fragment of HC1 which served as a negative control. **d)** Yeast 2-hybrid β-galactosidase activity in homogenates of yeast transformants co-expressing the LC1 COOH-terminal domain CT-LC1 (fused to the DNA-binding domain of the LexA protein) and various deletion mutants of α1-syntrophin as indicated, or a fragment of RACK-1 as negative control (*NC*; each fused to the transcription activation domain). β-galactosidase activity in Miller units is given in percent relative to activity obtained with the NH_2_ terminus of the MAP1B heavy chain which has been demonstrated previously to interact with the LC1 COOH-terminal domain [4] and was used here as positive control. The largest α1-syntrophin fragment containing the PH1b, PH2, and SU domains was identified in the original yeast 2-hybrid screen as interaction partner of LC1. Elevated levels of β-galactosidase activity were detected with all α1-syntrophin fragments containing the PH2 domain. Autoactivation of the α1-syntrophin deletion mutant proteins was excluded (data not shown). Two independent yeast colonies were assessed for each two-hybrid pair in 4 independent experiments. Values represent the mean ± standard deviation. **e)** The indicated recombinant α1-syntrophin fragments were subjected to SDS-PAGE, blotted onto nitrocellulose and either stained with amido black or probed with recombinant LC1 protein. For immunological detection of LC1 bound to blotted proteins anti-LC1 was used. LC1 was found to bind to syntrophin fragments containing the PH2 or PDZ domain, but not to GST which served as a negative control. Anti-LC1 did not directly react with the blotted proteins when prior incubation of the blot with LC1 was omitted (not shown).

The Matchmaker 2-hybrid system (Clontech, Mountain View, California) was employed following the manufacturer’s recommendations. Using the small scale transformation procedure, strain EGY48 (p8oplacZ) carrying a plasmid encoding the COOH terminus of LC1 (CT-LC1 in Fig. 1a, corresponding to MH3B [4]) fused to the DNA binding domain of the LexA protein was transformed with 500 µg of a cDNA library prepared from 19-day old mouse embryo and cloned into a vector resulting in fusions of the cDNA clones with the transcription activation domain of the Matchmaker system.

### Constructs

Constructs for use in yeast: Construction of the plasmid encoding the bait protein for the screen, the COOH terminus of rat MAP1B LC1 (CT-LC1 in Fig. 1a, corresponding to MH3B [4]) fused to the DNA binding domain of the 2-hybrid system, was described previously [4]. A mouse α1-syntrophin fragment comprising the PH1b, PH2, and SU domains (amino acids 172–503; Fig. 1a) fused to the transcription activator domain in vector pB42AD of the 2-hybrid system was obtained in the screen; this clone was used as template to generate α1-syntrophin deletion mutants by PCR such that the PCR fragments were amenable to restriction with *Eco*RI and *Xho*I to be inserted into the *Eco*RI and *Xho*I restriction sites of pB42AD, resulting in the respective α1-syntrophin domain fused to the transcription activator domain. The following deletion mutants were generated: PH2-SU, amino acids 284–503, using primers 5′-CCGGAATTCGGGAGCCAGGACATCAAGCAGATTGGC-3′ and 5′-GGTAGACAAGCCGACAACCTTGATTGGA-3′; PH2, amino acids 284–441, using primers 5′-CCGGAATTCGGGAGCCAGGACATCAAGCAGATTGGC-3′ and 5′-CCGCTCGAGCGGCTCGGGCTGCTCCAG-3′; SU, amino acids 433–503, using primers 5′-CCGGAATTCGCAGCTGAGCCTGGAGCAGCCCGAGCC-3′ and 5′-GGTAGACAAGCCGACAACCTTGATTGGA-3′. For a negative control (*NC*; Fig. 1d) we used a COOH-terminal fragment of murine RACK-1 (amino acids 173–317) fused to the transcription activator domain in vector pB42AD [31].

Constructs for the expression of recombinant proteins in *E. coli*: The construction of plasmids encoding 6xHis-tagged LC1, LC2, CT-LC1, and NT-HC1 (MH1B [4]) has been described [3], [4]. 6xHis-tagged full length α1-syntrophin was generated by PCR using primers 5′-CCGCAATTGATGGCGTCAGGCAGGCGC-3′ and 5′-GTCCCAGCCAACGGAGGTCCC-3′ and a mouse full length α1-syntrophin cDNA [32] as template. The PCR fragment was restricted with *Mun*I and *Eco*RI yielding a fragment comprising amino acids 1–172 of α1-syntrophin. This fragment was joined in a triple ligation to the *Eco*RI/*Xho*I fragment of the α1-syntrophin cDNA obtained in the 2-hybrid screen containing the rest of the coding sequence (amino acids 172–503) and a *Mun*I/*Xho*I restricted derivative of pQE-60 (Qiagen, Valencia, California) to yield construct pQEsyn encoding full length mouse α1-syntrophin with appropriate flanking restriction sites. The full length α1-syntrophin cDNA was cut out with *Mun*1 and *Xho*I and inserted into the *EcoR*I and *Xho*I sites of a derivative of pET15b (Novagen, Madison, Wisconsin) resulting in a construct encoding NH_2_-terminally 6xHis-tagged α1-syntrophin. Likewise, fragments containing α1-syntrophin domains PH2 plus SU (PH2-SU) or PH2 only were obtained by digestion with *Eco*RI and *Xho*I of the respective pB42AD vectors (see above) and inserted into the *Eco*RI and *Xho*I sites of the derivative of pET15b resulting in constructs encoding NH_2_-terminally 6xHis-tagged PH2-SU and PH2 domains, respectively. To express the PDZ domain of α1-syntrophin (amino acids 80–164), a PCR fragment containing the PDZ domain was generated using the full length α1-syntrophin cDNA (see above) as template and primers 5′-ACTGGAATTCCGCCGCGTGACGGTGCGCAAGGC-3′ and 5′-ATCGCTCGAGCTTCATGTACTTAACCTCCAACACAACCTCCTTGCCTGTCTTC-3′. The resulting PCR fragment was inserted by blunt end ligation into the *Eco*RV site of pBluescript (Fermentas, Glen Burnie, Maryland), cut out with *Eco*RI and *Xho*I and inserted into plasmid pGEX-4T-1 (GE Healthcare Biosciences AB, Uppsala, Sweden) to yield a construct for the expression of the NH_2_-terminally GST-tagged PDZ domain of α1-syntrophin.

For expression in vertebrate cells we used a construct encoding COOH-terminally myc-tagged LC1 described previously [5]. For the expression of α1-syntrophin we fused the *Mun*I/*Xho*I fragment of pQEsyn encoding full length α1-syntrophin (see above) in frame to NH_2_-terminal GFP in an *Eco*RI/*Xho*I restricted derivative of pEGFP-C1 (Clontech) which contains a human cytomegalovirus immediate early promoter.

The correct sequence of all constructs was confirmed.

### Antibodies

Affinity-purified rabbit polyclonal anti-LC1 antibody [5] and anti-HC1 antibody (anti-HC750) [33]; affinity-purified rabbit polyclonal anti-myc [5]; monoclonal pan anti-syntrophin antibody anti-syn1351 [34]; polyclonal rabbit anti-DRP2 [35] kindly provided by P. Brophy; polyclonal rabbit anti-paranodin antibody SL51 [36] kindly provided by J. A. Girault; polyclonal rabbit anti-ezrin (Upstate, Lake Placid, NY); monoclonal mouse anti-α-tubulin B-5-1-2 (Sigma, St. Louis, MO) and monoclonal rat anti-α-tubulin (YL1-2; Abcam, Cambridge, UK); Secondary antibodies: Alexa Fluor 488- or Fluor 568-labelled goat anti-rabbit, anti-rat, or anti-mouse (Molecular Probes, Leiden, The Netherlands), rhodamine-red-labeled goat anti-mouse or anti-rabbit (Jackson, West Grove, PA) and Cy5-labelled donkey anti-rat (Jackson). Secondary antibodies for immunoblots: HRP-conjugated goat anti-rabbit or anti-mouse (Jackson) or alkaline phosphatase-conjugated anti-rabbit or anti-mouse (Promega, Mannheim, Germany).

### Immunohistochemistry, Cell Culture, Transfection, and Microscopy

Wild-type and MAP1B−/− mice [13] were anesthetized and sacrificed by decapitation. Sciatic nerves were dissected and fixed with 4% PFA in PBS for 10 min at room temperature. After washing with PBS, nerves were teased on SuperFrost Plus glass slides (Thermo Fisher Scientific, Waltham, MA), air-dried and stained as described [37]. PtK2 cells were grown, transiently transfected, and stained for fluorescence microscopy with a confocal Zeiss Axiovert microscope with LSM 510 software (Zeiss, Oberkochen, Germany) as described [3].

### Protein Analysis

His-tagged recombinant proteins were expressed in E. Coli and purified as described [3].

### Protein Interaction Assay with Europium Labeled Proteins

Europium labeling of recombinant α-syntrophin and binding assays were performed as described previously [38]. Briefly, 96-well microtiter plates were coated with 100 nM LC1 or BSA type H1 (Gerbu, Gaiberg, Germany) as a control. Following blocking with 4% BSA, plates were overlaid with increasing amounts of Eu^3+^-labeled α1-syntrophin. Plates were washed and protein bound was determined by releasing the complexed Eu^3+^ with enhancement solution and measuring fluorescence with a Delfia time-resolved fluorometer (Wallac, Turku, Finland). Binding of α-syntrophin to BSA was considered to be non-specific.

For blot overlay assays, recombinant proteins were fractionated by SDS–PAGE. Blots (nitrocellulose membrane, 0.2 µm; Schleicher & Schuell, Dassel, Germany) were blocked in buffer A (0.25% Tween 20 in phosphate-buffered saline) containing 2% bovine serum albumin (BSA) for 1 h, washed 3 times for 5 min in buffer A, incubated with 10–100 µg/ml recombinant protein in buffer A containing 2% BSA for 2 h, washed again, and probed with an appropriate primary antibody against the recombinant protein in buffer A containing 1% BSA. After additional washing, the recombinant protein-antibody complexes were detected using alkaline phosphatase-conjugated secondary antibodies (Promega, Mannheim, Germany) and a detection system described previously [39] or horse radish peroxidase-conjugated secondary antibodies (Jackson, West Grove, Pennsylvania) and the chemiluminescence detection system (Pierce, Rockford, Illinois) according to the manufacturer’s recommendations.

### Immunoprecipitation

For immunoprecipitations, whole brains of 3-week-old or sciatic nerves of 6-day-old wild-type or transgenic mice expressing myc-tagged LC1 in the nervous system [40] were homogenized on ice in TEN buffer (100 mM Tris-HCl (pH 7.5), 100 mM NaCl, 10 mM EDTA, 0.1 mM DTT) containing a mixture of protease inhibitors (Roche, Vienna, Austria). Immunoprecipitates [5] were analyzed by immunoblotting as described [39].

### β-galactosidase Assay

Ortho-nitrophenyl-β-d-galactopyranoside was used as the substrate for β-galactosidase for the liquid culture assay (Clontech). For each two-hybrid pair, two independent yeast colonies were selected, grown to an OD_600_ of 0.5–0.8, harvested, resuspended in Z-buffer (60 mM Na_2_HPO_4_, 40 mM NaH_2_PO_4_, 10 mM KCl, 1 mM MgSO_4_, pH 7.0) with 0.27% (v/v) 2-mercaptoethanol, and lysed by vigorous shaking in a “Merkenschlager” cell mill using glass beads. Protein concentration was determined by the method of Bradford [41] and β-galactosidase activity was measured and calculated in modified Miller units: 1 U = 1000_*_OD_420_/t_*_V_*_mg protein. Values were expressed as percent relative to the activity obtained with the positive control reaction indicated.

## Results

### The Light Chains of MAP1B and MAP1A Interact with α1-syntrophin

The COOH-terminal domain of MAP1 proteins is conserved in all members of this protein family from drosophila to man. To identify proteins interacting with this conserved domain which is located in the light chains of mammalian MAP1A, MAP1B and MAP1S, we performed a yeast 2-hybrid screen using this domain of LC1 as bait and a mouse 19-day embryo cDNA library as target. One of the candidate proteins identified in this screen was α1-syntrophin, a modular adapter protein with multiple protein interaction motifs associated with the dystrophin protein family [15]–[18].

We first confirmed that the light chains of MAP1B and MAP1A directly interact with α1-syntrophin. Purified recombinant α1-syntrophin bound specifically to LC1 in a microtiter plate overlay assay (Fig. 1b). Likewise, in a blot overlay assay, recombinant α1-syntrophin bound to LC1, LC2, and the conserved COOH-terminal domain which was used as bait in the original screen (Fig. 1c). In contrast, α1-syntrophin did not interact with the NH_2_-terminal domain of MAP1B (Fig. 1c). This 508-amino acid domain is also conserved in all proteins of the MAP1 family and was used here as negative control.

To identify which domain(s) of α1-syntrophin interact with LC1 we first performed a yeast 2-hybrid β-galactosidase assay. Starting with the α1-syntrophin cDNA fragment that interacted with LC1 in the original screen and contained the PH1b, PH2, and SU domains we analyzed the interaction with LC1 of several α1-syntrophin deletion mutants. We found that the COOH terminus of LC1 (the bait protein of the screen) interacted with all α1-syntrophin deletion mutants that contained the PH2 domain (Fig. 1d), revealing that this domain contains an LC1 binding site. This interaction of LC1 with the PH2 domain was confirmed in blot overlay assays (Fig. 1e). Since α1-syntrophin also contains a PDZ domain and LC1 and LC2 have been reported to interact with PDZ domains of other proteins [40], [42] we tested interaction of LC1 with a recombinant protein containing the PDZ domain of α1-syntrophin fused to glutathione S-transferase (GST). In a blot overlay assay, LC1 interacted specifically with the PDZ domain but not with GST (negative control, Fig. 1e).

**Figure 2:**
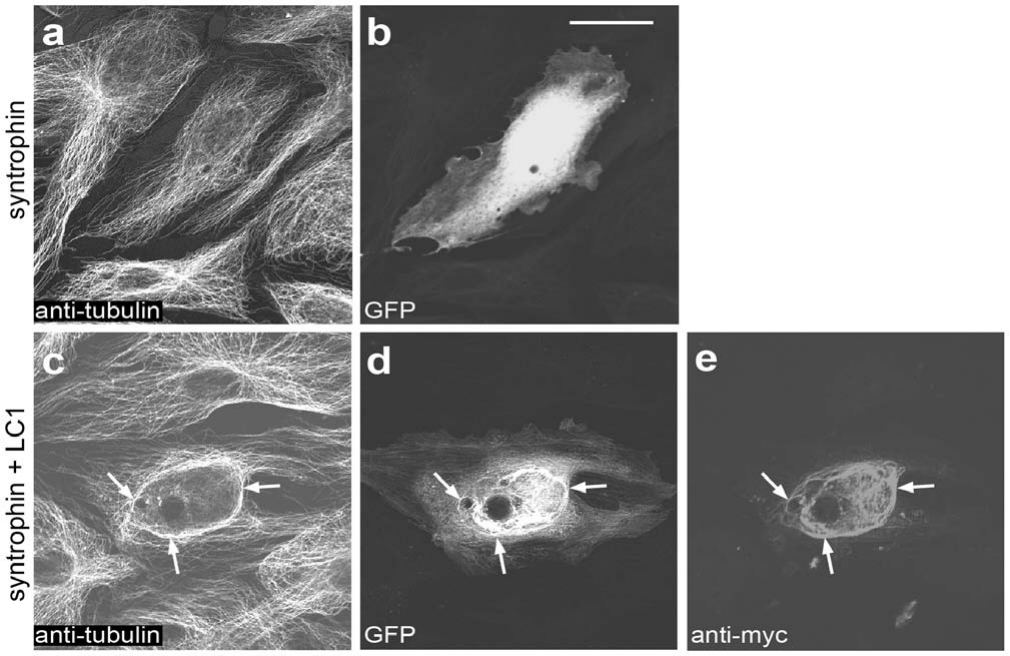
α1-syntrophin binds to microtubules in cells expressing LC1. PtK2 cells were transiently transfected to express either EGF-tagged α1-syntrophin alone (a and b) or α1-syntrophin and myc-tagged LC1 (c–e). Cells were fixed, co-stained for tubulin (*anti-tubulin*) and LC1 (*anti-myc*) and analyzed by fluorescence microscopy. In the absence of ectopically expressed LC1, α1-syntrophin was diffusely distributed throughout the cytoplasm (b). When co-expressed with LC1, α1-syntrophin was found to co-localize with LC1 on microtubules (c-e, arrows). Expression of LC1 causes microtubules to bundle, as has been described previously [5]. Scale bar, 20 µm.

To confirm cellular interaction of α1-syntrophin with LC1 we ectopically expressed tagged versions of the two proteins in PtK2 cells. In the absence of LC1, GFP-tagged α1-syntrophin displayed diffuse distribution (Fig. 2). We did not observe binding of α1-syntrophin to actin in epitheloid PtK2 cells as has been described for endothelial, smooth muscle, and Chinese hamster ovary cells [43]. Upon co-expression of LC1, α1-syntrophin was found to co-localize with LC1 on microtubules. The truncated version of α1-syntrophin comprising the PH1b, PH2, and syntrophin unique domains which bound to LC1 in vitro (Fig. 1) also interacted with LC1 in PtK2 cells (not shown). Cells expressing GFP only in the presence of LC1 displayed diffuse distribution of GFP, ruling out the possibility that the co-localization of α1-syntrophin with LC1 is due to the GFP-tag (not shown).

**Figure 3:**
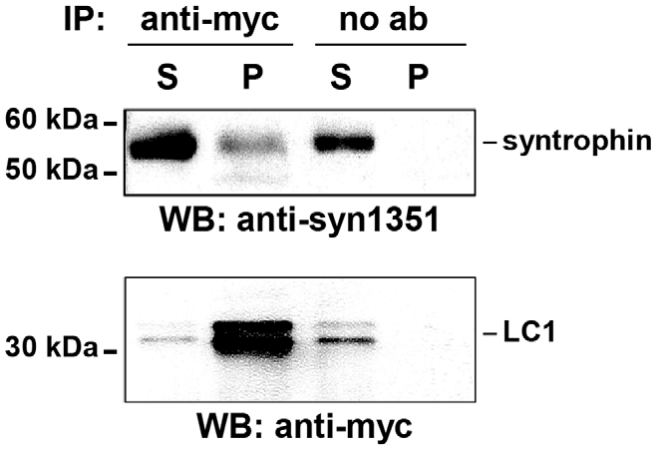
α1-syntrophin is found in a complex with LC1 in the central and peripheral nervous system. Brain protein extracts obtained from transgenic mice expressing myc-tagged LC1 were immunoprecipitated (*IP*) either with anti-myc antibodies (*anti-myc*) or without antibody (*no ab*; negative control). Pellets (*P*) and the corresponding supernatants (*S*) were fractionated by SDS-PAGE and analyzed by immunoblotting (*WB*) using anti-syntrophin (*anti-syn1351*) or anti-myc antibodies (*anti-myc*). The positions of protein size markers, syntrophin, and LC1 are indicated. The double band corresponding to LC1 resulted from insufficient denaturation prior to gel electrophoresis.

Further confirmation for the association of α1-syntrophin with LC1 in vivo was obtained by co-immunoprecipitation experiments. Using wild-type mouse brain extracts and anti-LC1 antibodies, a small amount of α1-syntrophin was co-precipitated (not shown). Since the epitope of the anti-LC1 antibody is located in the COOH-terminal part of LC1 that has been identified as interaction domain for α1-syntrophin (Fig. 1), binding of α1-syntrophin in this region might reduce interaction of anti-LC1 with the LC1-syntrophin complex. Therefore, we next used protein extracts of brains from three-week-old transgenic mice which express myc-tagged LC1 in the brain [40]. Using anti-myc antibodies, syntrophin was co-precipitated with myc-tagged LC1, demonstrating that the proteins associate with each other in vivo (Fig. 3a).

The above experiments clearly demonstrate that the light chains of MAP1B and MAP1A can directly interact with α1-syntrophin and associate with syntrophin in the nervous system in vivo. Furthermore, we localized the light chain interaction sites to the PH2 and PDZ domains of α1-syntrophin.

### MAP1B and Syntrophin Co-localize in Schwann Cells in Adult Peripheral Nerve

**Figure 4:**
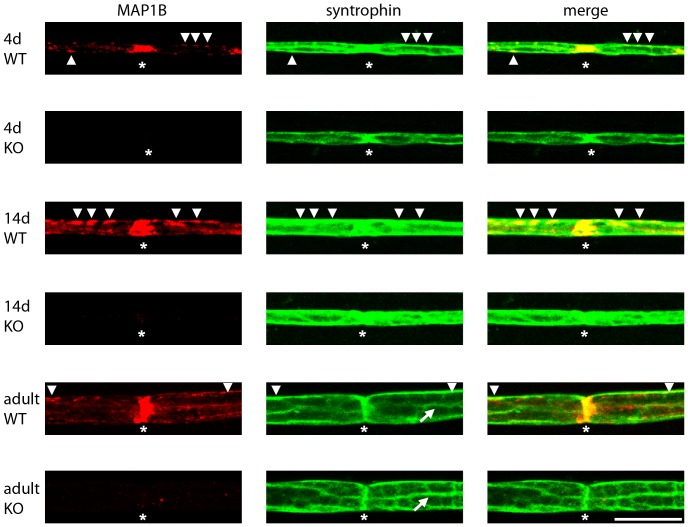
MAP1B and syntrophin co-localize at the nodes of Ranvier and the abaxonal Schwann cell membrane. Sciatic nerves were prepared from 4-day (*4d*) or 14-day (*14d*) old or adult (*adult*) wild-type (*WT*) and MAP1B^−/−^ (*KO*) mice. Individual myelinated axons were isolated and stained for MAP1B (antibody anti-HC750) or syntrophin (pan syntrophin antibody anti-syn1351) as indicated. The pictures represent projections of confocal Z-stacks. The staining for MAP1B in postnatal and adult Schwann cells is specific as it is absent in Schwann cells of MAP1B^−/−^ mice. At all ages MAP1B was found to be concentrated at the nodes of Ranvier (*asterisks*). It also localized at the abaxonal membrane (*arrow heads*), particularly strong at postnatal day 14. Syntrophin was also found at nodes of Ranvier and the abaxonal membrane. In the adult, it was found to be localized to Cajal bands (*arrows*) in agreement with previous results [44]. Co-localization of MAP1B and syntrophin was most prominent at the nodes of Ranvier and partial co-localization was found at the abaxonal membrane (*arrow heads*). Scale bar, 20 µm.

We next analyzed the subcellular localization of MAP1B and syntrophin in peripheral nerve by staining teased fibers of sciatic nerves of wild-type and MAP1B deficient mice at different stages during postnatal development (Fig. 4). At all ages tested (4 days, 14 days, and adult) MAP1B appeared to be highly concentrated at the nodes of Ranvier (Fig. 4). Specific MAP1B staining was also observed in the abaxonal Schwann cell membrane. At postnatal day 14 this staining was particularly prominent. Syntrophin was found at the nodes of Ranvier and in the abaxonal membrane. At both locations it partially co-localized with MAP1B. Confirming previous results, we observed that, in the adult, syntrophin was localized at Cajal bands [44]. Teased fibers of wild-type and MAP1B knockout mice did not display consistent differences in syntrophin localization (Fig. 4).

**Figure 5:**
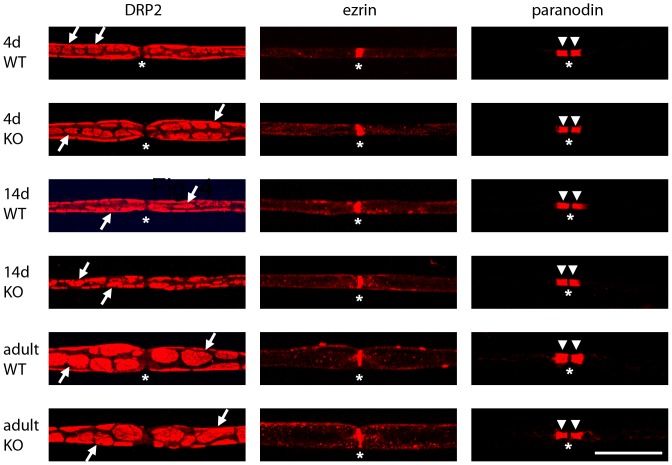
DRP2, ezrin and Caspr1/paranodin localization in peripheral nerve is not affected by MAP1B deficiency. Sciatic nerves were prepared from 4-day (*4d*) or 14-day (*14d*) old or adult (*adult*) wild-type (*WT*) and MAP1B^−/−^ (*KO*) mice. Individual myelinated axons were isolated and stained for DRP2, ezrin, or Caspr1/paranodin as indicated. The pictures represent projections of confocal Z-stacks. The nodes of Ranvier (*asterisks*) and the DRP2 clusters (*arrows*) are indicated where it was possible to define them. The gap in Caspr1/paranodin staining at the node is due to the absence of the protein in the actual node region which is flanked by a proximal and a distal paranodal compartment (*arrow heads*). Scale bar, 20 µm.

We also analyzed whether other Schwann cell proteins such as DRP2 and ezrin and the axonal protein Caspr1/paranodin were affected by deficiency in MAP1B (Fig. 5). During postnatal development and in the adult, DRP2 was expressed in characteristic clusters at the abaxonal Schwann cell membrane [35]. Ezrin was found to be expressed in defined regions which sharpened during postnatal development. These regions represent Schwann cell microvilli at the nodes of Ranvier [45]. Caspr1/paranodin marked the paranodal axonal compartments [36], [46] at early and later stages (Fig. 5). No differences in DRP2, ezrin, or Caspr1/paranodin staining were observed between wild-type and MAP1B deficient fibers during postnatal development and in the adult. Moreover, no significant differences could be detected between wild-type and MAP1B deficient fibers in the distance between consecutive nodes of Ranvier (normalized to fiber diameter), in the number and spacing of Schmidt-Lantermann incisures, or in the distance from the nodes of Ranvier to the first Schmidt-Lantermann incisure in the internodal region (not shown).

## Discussion

Our results demonstrate that the light chains of MAP1A and MAP1B interact with the modular adapter protein α1-syntrophin in the central and peripheral nervous system. We identified the conserved COOH termini of the light chains and the PH2 and PDZ domains of syntrophin as interacting domains. The light chains of MAP1A and MAP1B have previously been reported to bind to PDZ domains of glutamate receptor interacting protein 1 [47], PDZrhoGEF [42], and neuronal nitric oxide synthase [40]. Thus, interaction with PDZ domains of target proteins involved in signal transduction emerges as a characteristic function of the COOH-terminal domain of the light chains of MAP1 proteins.

The direct comparison of MAP1B expression in sciatic nerve fibers of wild-type and MAP1B−/− mice by immunohistochemistry allowed us to specifically detect MAP1B expression in myelinating Schwann cells of the adult, uninjured nerve and at higher levels in Schwann cells during postnatal development (Fig. 4). MAP1B was found at the nodes of Ranvier and at the internodal abaxonal membrane. At both locations MAP1B partially co-localized with syntrophin. Syntrophins including the α-, β1-, β2- and γ2-isoforms have previously been shown to be present in the internodal abaxonal Schwann cell membrane, in particular localizing to the Cajal bands where they confer specific scaffolding properties to the dystrophin glycoprotein complex [44]. In the present study we used a pan syntrophin antibody reacting with α-, β and γ-isoforms for the analyses in peripheral nerve (Fig. 3 and 4). Thus, while our biochemical analysis (Fig. 1) clearly shows that the light chains of MAP1A and MAP1B interact with α1-syntrophin, we cannot exclude the possibility that the light chains also interact with other isoforms of syntrophins in the peripheral nerve.

MAP1B deficiency did not appear to alter syntrophin expression or localization in Schwann cells, nor did it alter the characteristic organization of DRP2 in clusters at the abaxonal Schwann cell membrane, nor the internodal distance (not shown) or the organization of the nodes of Ranvier as analyzed by staining for ezrin and Caspr1/paranodin (Fig. 5). Thus, the previously observed reduction in nerve conductance velocity and the reduced myelination in MAP1B deficient sciatic nerves [13] do not appear to be reflected by changes in syntrophin expression or intracellular organization.

Originally, MAPs were thought to regulate neuronal microtubule dynamics, stability, and spacing between individual microtubules in a microtubule bundle [1] as well as modulating access and activity of microtubule-dependent motor proteins and thus axonal and dendritic transport [48]–[55] through their direct interaction with microtubules. In a more recent development, classical MAPs have been found to bind to a wide variety of proteins with diverse functions. For example, proteins of the MAP1 family bind to receptors and ion channels [56]–[58], postsynaptic density (PSD) proteins PSD-93 and PSD-95 [59], [60], signaling molecules (EPAC, PDZrhoGEF, Tiam1, casein kinase 1d, RASSF1a [42], [61]–[66]) and proteins involved in intracellular traffic [67]–[70]. Thus, our findings presented here add to a growing body of evidence that classical MAPs can play a role in signal transduction not only by directly modulating microtubule function, but also through their interaction with a variety of signal transduction proteins.
